# Three State Estimation Fusion Methods Based on the Characteristic Function Filtering

**DOI:** 10.3390/s21041440

**Published:** 2021-02-19

**Authors:** Yiran Yuan, Chenglin Wen, Yiting Qiu, Xiaohui Sun

**Affiliations:** 1 Institute of Systems Science and Control Engineering, School of Automation, Hangzhou Dianzi University, Hangzhou 310018, China; yuanyiran@hdu.edu.cn (Y.Y.); 191060063@hdu.edu.cn (Y.Q.); sun_xh@163.com (X.S.); 2 School of Automation, Guangdong University of Pertrochemical Technology, Maoming 525000, China

**Keywords:** characteristic function, multi-sensor, fusion method, parallel filtering, sequence filtering

## Abstract

There are three state estimation fusion methods for a class of strong nonlinear measurement systems, based on the characteristic function filter, namely the centralized filter, parallel filter, and sequential filter. Under ideal communication conditions, the centralized filter can obtain the best state estimation accuracy, and the parallel filter can simplify centralized calculation complexity and improve feasibility; in addition, the performance of the sequential filter is very close to that of the centralized filter and far better than that of the parallel filter. However, the sequential filter can tolerate non-ideal conditions, such as delay and packet loss, and the first two filters cannot operate normally online for delay and will be invalid for packet loss. The performance of the three designed fusion filters is illustrated by three typical cases, which are all better than that of the most popular Extended Kalman Filter (EKF) performance.

## 1. Introduction

In practical, filtering methods play an important role in state estimation, such as fault diagnosis, target tracking, signal processing, computer vision, communication, navigation, and other fields [1]. The traditional Kalman Filter (KF) has several good advantages, such as real-time, recursive, and optimal. It is only suitable for linear systems and Gaussian white noises [2]. However, these conditions are difficult to meet in the actual situation. In 1961, Bucy established a filtering method for nonlinear system based on Taylor expansion, called as Extended Kalman Filter (EKF); it was established by the first-order linear approximation to convert it to the standard KF form [3,4]. However, as the nonlinearity increases, the performance gradually decreases. In 1995, Julier established the Unscented Kalman Filter (UKF) based on the unscented transformation [5,6]. In 2009, Arasaratnam and Haykin established the Cubature Kalman Filter (CKF) based on the approximation of points [7,8]. Both the UKF and CKF use sigma interpolation to design a form similar to Kalman filter, so as to improve the influence caused by truncation error, and can achieve the second-order approximation [9,10,11,12]. However, no matter EKF, UKF, or CKF, it still cannot show better performance in strongly nonlinear systems.

When the modeling error of the system is described by the density function, Gordon et al. developed the particle filter (PF), employing the density function of the error as the objective function [13,14]. The PF can solve general non-Gaussian problems [15]. However, since PF is based on conditional probability density, the implementation of PF relies on a large number of particle samplings, which makes the calculation complexity high [16]. The degradation of particles during the resampling process will reduce the speed and accuracy of filters. The later-developed Ensemble Kalman Filter (EnKF) improved the computational complexity of high-dimensional [17], Maximum Correlation Entropy Kalman Filter (MCKF) canceled the requirement for white noise [18], etc., but they still cannot achieve the desired effect in the strong nonlinear system. Therefore, the design of Kalman-style filters that can be applied to stronger nonlinear systems has always received widespread attention, but there are still no breakthrough results.

In order to improve the applicability of Kalman-form filters in strongly nonlinear systems, for a class of systems with liner state and strongly nonlinear measurement, Zhou uses the characteristic function to replace the probability density function to design a characteristic function filter (CFF) [19]. Since the strong nonlinear measurement model is not required to be changed, therefore, large truncation or approximation errors caused by serious changes in measurement equations such as EKF, UKF, and CKF are avoided. However, its application filed is only limited in one-dimensional models. In Reference [20], Wen designed a filter with general dimensions by establishing new performance index. At the same time, by linearizing the state model by Taylor expansion, CFFs have been applied to a system whose state is weakly nonlinear and the measurement is strong nonlinearity [21].

The CFF, for strong nonlinear measurement system, has three typical application directions. First, it has been successfully applied when the state model is linear [20]; in other examples, such as in space target tracking, the state model is established in the Cartesian coordinate system, and the measurement model is based on the polar coordinate system, the relationship between the state and the measurement is super nonlinear. Another possible application in network parameter solving, if the model parameter to be identified is regarded as a linear state model of random walk, and the neural network model as the measured value is a super nonlinear function with the parameter to be determined as the variable. Second, the state model that is weakly nonlinear has also been successfully applied [21]. This situation is common in industrial systems. Third, the state model with strongly nonlinear is a problem that needs to be solved, mainly because the linearization process of the model will cause the greatest loss of information.

While the measured or recognized target has multiple attributes or is interfered by multiple uncertain factors, it has to employ multiple sensors to complete a common detection task. In a complex large-area environment, the tracking of a non-cooperative target needs to be completed by sensors distributed in different places, since the relationship between the state and the measurement is super nonlinear, and each sensor model is also strongly nonlinear. Since each sensor and the fusion center are usually connected wirelessly, it is necessary to design fusion methods with different requirements to coordinate the tracking task of the target. Under ideal transmission, it is necessary to design a high-precision fusion filter. If the requirements for real time performance are also additionally considered, a fast running fusion filter needs to be designed. When there exist delay and packet loss in the transmission process, it is necessary to design a corresponding fusion filter, etc. However, the research result about these fusion filters based on CFF is not found.

**Figure 1:**
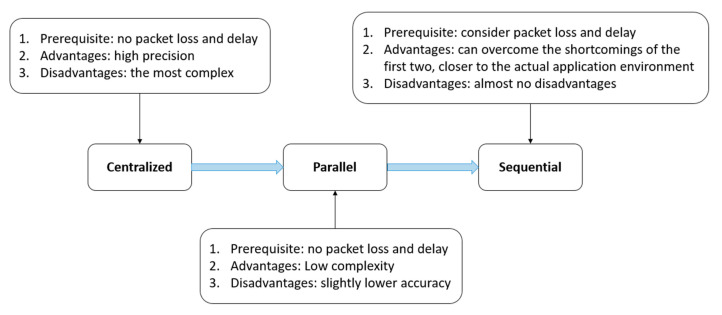
Illustration of three fusion methods.

Consequently, to meet accuracy requirements, the centralized filter will be designed; To consider real time, parallel filter will be designed; To meet the needs of network packet loss and delay, sequential filter will also be designed. The prerequisites, advantages, disadvantages, and the relationship between the three fusion methods are shown in Figure 1.

The main arrangement of this paper is as follows: Section 1 offers the background introduction; Section 2 establishes the model; Section 3, Section 4 and Section 5 give a description of three fusion methods; Section 6 offers the detailed implementation process of sequential fusion method; Section 7 discusses the simulation of typical cases; and Section 8 offers the method summary and outlook.

## 2. Problem Description

**Figure 2:**
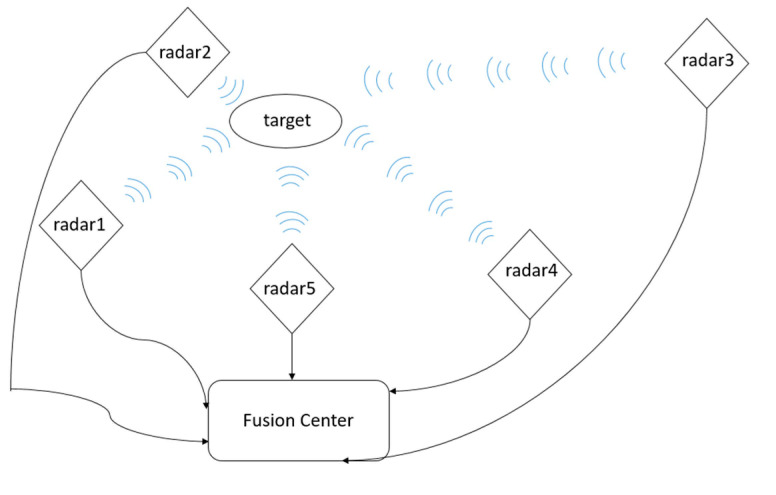
Model building flowchart.

Consider a type of dynamic system whose state is linear and measured by several sensors [22]. For example, for a moving target in larger space area, there are multiple radars on the ground, distributed in different places, to simultaneously observe the target. The distribution between each radar and the target is far or near, and the transmission of information may be fast or slow. Moreover, after the radar collects the information, it needs to be transmitted to the fusion center for data processing, as shown in Figure 2.

Therefore, a multi-dimensional state space model is established as follows:

(1) X(r)=Γ(r,r−1)X(r−1)+λ(r,r−1)w(r−1)

(2) $$Y_{i}(r)=h_{i}(X(r))+v_{i}(r)\;$$

where $X(r)\in R^{n\times 1}$ and $Y_{i}(r)\in R^{m_{i}\times 1}$ are state vector and measurement vector respectively; w(r) and $v_{i}(r)$ are system noises; Γ(r,r−1) is the state transition matrix; λ(r,r−1) is the known process drive noise; and $h_{i}(\cdot )$ is a continuous smooth nonlinear function [23]. Moreover, i=1,2,⋯,N represents the number of sensors.

The measurement model of Equation (2) can be rewritten as a concise form, as follows:

(3) $$Y(r)={\left[\begin{array}{cccc} Y_{1}^{}(r) & Y_{2}^{}(r) & \cdots & Y_{N}^{}(r) \end{array}\right]}^{T}$$

(4) $$h(X(r))={\left[\begin{array}{cccc} h_{1}^{}(X(r)) & h_{2}^{}(X(r)) & \cdots & h_{N}^{}(X(r)) \end{array}\right]}^{T}$$

(5) $$v(r)={\left[\begin{array}{cccc} v_{1}^{}(r) & v_{2}^{}(r) & \cdots & v_{N}^{}(r) \end{array}\right]}^{T}$$

Correspondingly, the multiple nonlinear measurement models in Equation (2) become the following:

(6) Y(r)=h(X(r))+v(r)

where $Y(r)\in R^{m}$, $m=m_{1}+m_{2}+\cdots +m_{N}$.

## 3. Centralized Characteristic Function Fusion Filtering Method under Multi-Dimensional Observation

### 3.1. Centralized Characteristic Function Filter Design

On the basis of the Equations (1) and (6), design filters in the form of Equations (7) and (8) are as follows:

(7) $$\hat{X}(r|r)=\Gamma (r,r-1)\hat{X}(r-1|r-1)+K(r)[Y(r)-\hat{Y}(r|r-1)]$$

(8) $$\hat{Y}(r|r-1)=h(\hat{X}(r|r-1))\;,$$

where $\hat{X}(r|r)\in R^{n\times 1}$ and $\hat{Y}(r|r-1)\in R^{m\times 1}$ are state estimate vector and measurement prediction vector respectively. $K(r)\in R^{n\times m}$ is the filter gain matrix to be designed.

### 3.2. Establishment of Error Characteristic Function Propagation Equation

According to Equations (1) and (7), we get the following:

(9) $$\gamma (r-1)=X(r-1)-\hat{X}(r-1|r-1)$$

where γ(⋅), X(⋅), and $\hat{X}(\cdot )$ represent the state error, the actual state value, and the state estimate value, respectively. By combining Equations (1), (7), and (9), the error recurrence equation can be obtained:

(10) $$\gamma (r)=\Gamma (r,r-1)\gamma (r-1)+\lambda (r,r-1)w(r-1)-K(r)[Y(r)-\hat{Y}(r|r-1)]$$

In Equation (10), note the following:

(11) s(r−1)=Γ(r|r−1)γ(r−1)

(12) q(r−1)=λ(r|r−1)w(r−1)

(13) $$\tilde{Y}(r|r-1)=Y(r)-\hat{Y}(r|r-1)$$

Then, Equation (10) can be simplified as follows:

(14) $$\gamma (r)=s(r-1)+q(r-1)-K(r)\tilde{Y}(r|r-1)$$

**Remark** **1.** *When Γ(⋅), λ(⋅), $\tilde{Y}(\cdot )$, and K(⋅) are all given, In Equation (14), we can see that γ can be represented by s(⋅), q(⋅), and a $K(\cdot )\tilde{Y}(\cdot )$. That is, the probability density function of γ is the conditional probability density function that depends on s(⋅) and w(⋅).*

In probability theory, the characteristic function of any random variable has been clearly given to completely define its probability distribution. Therefore, on the basis of KF, replace the probability density with characteristic function, and a new form of CFF is used to study the fusion algorithm.

For any random variable X, we denote its characteristic function as $\psi_{X}(t)$, which is defined as follows:

(15) $$\psi_{X}(t)=E(e^{itX})$$

where, t represents any real number, and E represents the expected value. 

In Equation (15), the right side of the equation is given by the Riemann–Steelches integral:

(16) $$E(e^{itX})=\int_{-\infty }^{\infty }e^{itx}dF_{X}(x),$$

where $F_{X}(x)$ is the distribution function of random variable X.

If the probability density function of the random variable X exists, Equation (16) can be further written as follows:

(17) $$E(e^{itX})=\int_{-\infty }^{\infty }e^{itx}f_{X}(x)dx,$$

where $f_{X}(x)$ is the probability density function of random variable X.

In Reference [19], two lemmas of characteristic function are given:

**Lemma** **1.** *Assuming multidimensional vector $x\in R^{n}$, $z\in R^{n}$ and $\psi_{z}(x)$ is the characteristic function of the strict system output. Define X=Az+b, where $A\in R^{m\times n}$, $b={\left[b_{1},b_{2},\cdots ,b_{m}\right]}^{T}$; The characteristic function expression of random variable X is $\psi_{X}(t)$, $t\in R^{m}$. Then the characteristic function of X can be expressed as follows.*

(18) $$\psi_{X}(t)=E\{e^{jtX}\}=E\{e^{jt(Az+b)}\}=e^{jtb}E\{e^{j(tA)z}\}=e^{jtb}\psi_{z}(tA)=\sum_{i=1}^{m}e^{jtb_{i}}\psi_{z}(tA)$$

**Lemma** **2.** *For two independent random variables, $X_{1}$ and $X_{2}$, let $X=X_{1}+X_{2}$, $X_{1},X_{2}\in R^{n}$, and then we get the following:*

(19) $$\psi_{X}(t)=\psi_{X_{1}+X_{2}}(t)=E\{e^{jt(X_{1}+X_{2})}\}=E\{e^{jtX_{1}}\}E\{e^{jtX_{2}}\}=\psi_{X_{1}}(t)\psi_{X_{2}}(t)$$

Usually, it is more complicated to directly use the probability density function to solve the analytical solution of the K(r) [24]. Here, we use the characteristic function to replace the probability density function to solve Equation (14). First, combine the definition of characteristic function and Equation (18), take the probability density function on both sides of Equation (14) at the same time, and then take the characteristic function at the same time, and we can get the following:

(20) $$\psi_{p_{\gamma (r)}}(t)=\psi_{p_{s(r-1)+q(r-1)-K(r)\tilde{Y}(r|r-1)}}(t)\;,$$

where p means probability density function, and ψ means characteristic function.

Combining Lemmas 1 and 2, Equation (20) becomes the following:

(21) $$\sum_{i=1}^{m}e^{jt\sigma_{i}}p_{\gamma (r)}=\left[\sum_{i=1}^{m}e^{jt\sigma_{i}}p_{s(r-1)}\right]\left[\sum_{i=1}^{m}e^{jt\sigma_{i}}p_{q(r-1)}\right]e^{-jtK(r)\tilde{Y}(r|r-1)}$$

By combining Lemmas 1 and 2 and Equation (21), the characteristic function propagation equation of the error recurrence equation is obtained:

(22) $$\psi_{\gamma (r)}(t)=\psi_{s(r-1)}(t)\psi_{q(r-1)}(t)e^{-jtK(r)\tilde{Y}(r|r-1)}$$

### 3.3. Establishment of Filter Performance Index 

It is pointed out in References [20,25] that K-L divergence can be used to quantify the difference between two different probability distributions. On the premise that the conditional estimation error characteristic function is obtained, and an objective function is given, the filter design can be carried out by describing the difference between the conditional estimation error characteristic function and the objective function. Therefore, the performance index of the characteristic function filter is designed as follows:

(23) $$M(r)=M_{0}(r)+K^{T}(r)R(r)K(r)$$

where M(r) represents the filter performance index, $M_{0}(r)$ is the parameter, R(r) is a positive definite weight matrix, and K(r) is the gain matrix to be estimated.

In Equation (23), let the following be [21]:

(24) $$M_{0}(r)=\left(\int \Lambda (t)\psi_{g}(r)\log\frac{\psi_{g}(t)}{\psi_{\gamma (r)}(t)}dt\right){\left(\int \Lambda (t)\psi_{g}(t)\log\frac{\psi_{g}(t)}{\psi_{\gamma (r)}(t)}dt\right)}^{T}$$

where Λ(t) represents the weight function, $\psi_{g}(t)$ is the characteristic function of the objective, and $\psi_{\gamma (r)}(t)$ is the characteristic function of the error.

**Remark** **2.** *In Equation (24), Λ(t) is introduced to ensure that $M_{0}(r)$ is bounded. Because $M_{0}(r)$ is a parameter, in order to ensure that it is non-negative, a transpose is multiplied to the right-hand side in Equation (24). For example, when the target is measured, the distance is non-negative, and the solution to the radial distance is in the form of a square, so the non-negativity of the parameters is guaranteed by multiplying by a transpose. The term log in Equation (24) can be understood as information entropy. The closer the values of $\psi_{g}(t)$ and $\psi_{\gamma (r)}(t)$ are, the better the performance of the filter. Moreover, in Equation (8), the measurement model of the system is multi-dimensional, so the objective function is in the form of a matrix. Then in Equation (23), M(r) is a multi-dimensional form, so the gain matrix K(r) is also multi-dimensional.*

### 3.4. Establishment of Equation for Solving Filter Gain Matrix K(r)

Bring Equation (22) into Equation (24), and then expand Equation (24). In the expanded Equation (24), let the following be:

(25) $$\begin{array}{ll} \sigma (r-1) & =\int_{}\Lambda (t)\psi_{g}(t)\log\psi_{g}(t)dt-\int_{}\Lambda (t)\psi_{g}(t)\log\psi_{s(r-1)}(t)dt\\ & -\int_{}\Lambda (t)\psi_{g}(t)\log\psi_{q(r-1)}(t)dt \end{array}$$

(26) $$\delta (r-1)=\int_{}\Lambda (t)\psi_{g}(t)jtdt$$

Combining Equations (23)–(26), Equation (23) can be rewritten as follows:

(27) $$\begin{array}{ll} M(r)= & \sigma (r)\sigma^{T}(r)+\sigma (r){\tilde{Y}}^{T}(r|r-1)K^{T}(r|r-1)\delta^{T}(r)+\delta (r)K(r)\tilde{Y}(r|r-1)\sigma^{T}(r)\\ & +\delta (r)K(r)\tilde{Y}(r|r-1){\tilde{Y}}^{T}(r|r-1)K^{T}(r)\delta^{T(r)}+K^{T}(r)R(r)K(r) \end{array}$$

By solving the first-order partial derivative of Equation (27) and taking it to be zero, we have the following:

(28) $$\frac{\partial M(r)}{\partial K(r)}=\delta^{T}(r)\sigma (r){\tilde{Y}}^{T}(r|r-1)+\delta (r)\delta^{T}(r)K(r)\tilde{Y}(r|r-1){\tilde{Y}}^{T}(r|r-1+2R(r)K(r)=0\;,$$

Then, taking the second-order partial derivative of Equation (27), we have the following:

(29) $$\frac{\partial^{2}M(r)}{\partial K^{2}(r)}=\delta^{T}(r)\delta (r)\tilde{Y}(r|r-1){\tilde{Y}}^{T}(r|r-1)+2R(r)>0$$

In Equation (29), when the second-order partial derivative is greater than zero, the solution of K(r) in Equation (28) is the minimum value. Bring the concentrated gain matrix K(r) into Equation (7), and the obtained estimated value is the optimal estimated value of the centralized fusion method.

**Remark** **3.** *In Equation (29), since $\delta (\cdot )\in R^{n\times 1}$, $\tilde{Y}(\cdot )\in R^{m\times 1}$, obviously $\delta^{T}(\cdot )\delta (\cdot )>0$ and $\tilde{Y}(\cdot ){\tilde{Y}}^{T}(\cdot )>0$. Since R(⋅) is positive definite, the second-order partial derivative of Equation (29) is greater than zero. At this time, the filter gain matrix obtained by Equation (28) is the optimal solution under the minimized performance index M.*

## 4. Parallel Characteristic Function Fusion Filtering under Multi-Dimensional Observation

### 4.1. Parallel Filter Design 

When the distribution position of each sensor and the distance from the fusion center is different, the centralized use of these sensors will increase the communication cost [26]. Moreover, as the number of sensors, N, increases, the filtering iteration process will become more complicated. Therefore, a class of distributed parallel filters is designed. For each sensor, there are the following:

(30) $$\hat{X}(r|r)=\Gamma (r,r-1)\hat{X}(r-1|r-1)+K_{i}(r)[Y_{i}(r)-{\hat{Y}}_{i}(r|r-1)]$$

(31) $${\hat{Y}}_{i}(r)=h_{i}(\hat{X}(r|r-1))$$

where i represents the *i*th sensor. Based on a network composed of multiple distributed sensors, design a parallel characteristic function filter as shown in Equation (32).

(32) $$\hat{X}(r|r)=\Gamma (r,r-1)\hat{X}(r|r-1)+\left[\begin{array}{ccc} K_{1}(r) & \cdots & K_{N}(r) \end{array}\right]\left[\begin{array}{c} Y_{1}(r)-{\hat{Y}}_{1}(r|r-1)\\ \vdots \\ Y_{N}(r)-{\hat{Y}}_{N}(r|r-1) \end{array}\right]$$

**Figure 3:**
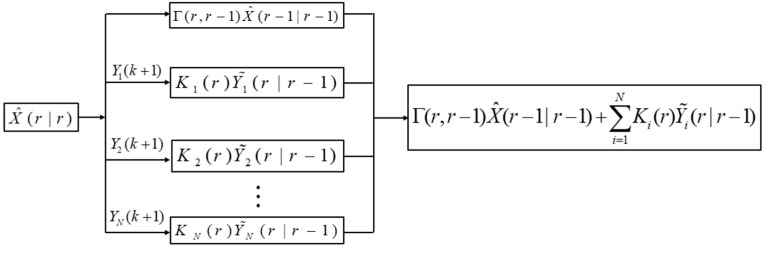
Parallel fusion filtering process.

The parallel fusion filtering process is shown in Figure 3.

### 4.2. Solve Each Gain Matrix $K_{i}(r)$ in the Parallel Filter Group

Follow and repeat the process of Section 3.2, Section 3.3 and Section 3.4. This subsection establishes an iterative numerical solution algorithm for the gain matrix, $K_{i}(r)$, based on the fixed point principle [27,28]. Since the numerical iterative solution method in this paper is based on the fixed point equation, if the problem of solving a certain equation can be converted into a fixed point problem, then it can be solved numerically. The fixed point is the point where D(c)=c. That is, for any equation D(c), if there is D(c)=c, then the fixed point equation of D(c) can be written in the form of c=d(c). Synthesize the above analysis, combined with Equation (28), and construct a fixed point equation $K_{i_{t}}(r)=d(K_{i_{t}}(r))$, to iteratively solve the $K_{i}(r)$:

(33) $$\begin{array}{ll} K_{i_{t+1}}(r)= & R_{i}^{-1}(r)\delta_{i}^{T}(r)\sigma_{i}(r){\tilde{Y}}_{i}^{T}(r|r-1)\\ & +R_{i}^{-1}(r)\delta_{i}^{}(r)\delta_{i}^{T}(r)K_{i_{t}}(r){\tilde{Y}}_{i}(r|r-1){\tilde{Y}}_{i}^{T}(r|r-1) \end{array}$$

where $t=0,1,2,\cdots ,T_{i}(r)$ represents the iteration steps. Therefore, based on the ith group of sensors, the gain matrix, $K_{i}(r)$, of its characteristic function filter is obtained, which is substituted into the parallel filter Equation (32), and the filter in the form of Equation (34) is obtained.

(34) $$\hat{X}(r|r)=\hat{X}(r|r-1)+\sum_{i=1}^{N}K_{T_{i}(r)}^{}(r)[Y(r)-{\hat{Y}}_{i}(r|r-1)]$$

The estimated value obtained at this time is the optimal solution of the parallel fusion method.

## 5. Sequential Characteristic Function Fusion Filtering under Multi-Dimensional Observation 

**Figure 4:**
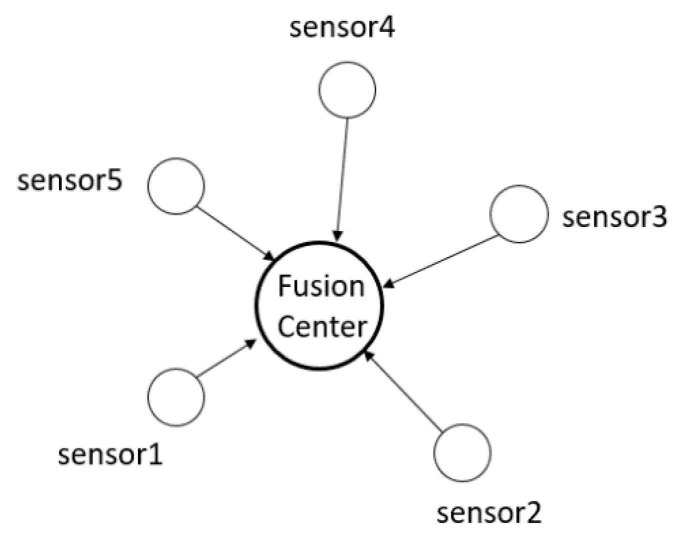
Schematic diagram of sensor fusion.

Considering that the distance between the sensor and the fusion center is different, the problem of transmission data delay and even packet loss due to network bandwidth constraints will occur. Then, on the basic of Equation (2), considering the reasons for network delay and packet loss, the measured value of the sensor data transmitted to the fusion center through the wireless network is marked as follows and the sensor fusion process is shown in Figure 4 [29]:

$$Y_{j_{1}}(r),Y_{j_{2}}(r),\cdots ,Y_{j_{i}}(r),\cdots ,Y_{j_{L}}(r)\;L\leq N$$

$$\{j_{1},j_{2},\cdots ,j_{i},\cdots ,j_{L}\}\subseteq \{1,2,\cdots ,i,\cdots ,N\}\;L\leq N$$

where, N means that we share N sets of sensors for measurement; L is the number of sensors that transmit data to the fusion center, after taking into account packet loss; and $j_{i}$ is the order in which the sensors arrive at the fusion center.

**Remark** **4.** *In this paper, we divide packet loss into two types. One is the data not transmitted to the fusion center in time, and the other is indeed lost data.*

### 5.1. Sequential Filter Design 

Considering the phenomenon of packet loss, it is assumed that the order of the measurement from each sensor arrives at the fusion center is 1,2,⋯,L (L≤N). Design a sequential characteristic function fusion filter as follows:

(35) $${\hat{X}}_{j_{i}}(r|r)={\hat{X}}_{{\left(j-1\right)}_{i}}(r|r)+K_{j_{i}}(r)[Y_{j_{i}}(r)-{\hat{Y}}_{j_{i}}(r|r-1)]$$

(36) $${\hat{Y}}_{j_{i}}(r)=h_{j_{i}}({\hat{X}}_{{\left(j-1\right)}_{i}}(r|r))$$

where $Y_{j_{i}}(r)\in R^{m_{j_{i}}\times 1}$, $K_{j_{i}}(r)\in R^{n\times m_{j_{i}}}$, and $j_{i}$ indicate that the order of group i sensors reaching the fusion center is j.

**Remark** **5.** *The sequential consideration of the order of information arrival, and first come first fusion. So use $j_{i}$ to indicate that the data collected by the i-th sensor is the j-th transmission to the fusion central. Moreover, $m_{j_{i}}$ represents the dimensionality of the observation value of the i-th sensor. Because the observation is multi-dimensional and the number of sensors is also multiple, thus the observation value of each sensor is also multi-dimensional. In order to add distinguish and clearly describe, we use $m_{j_{i}}$ to represent it.*

### 5.2. The Establishment of Error Recurrence Equation 

Follow and repeat the process of Section 3.2, to get the following:

(37) $$\gamma_{{\left(j-1\right)}_{i}}(r)=X_{{\left(j-1\right)}_{i}}(r)-{\hat{X}}_{{\left(j-1\right)}_{i}}(r|r)\;,\;j=1,2,\cdots ,L$$

Then the error recurrence equation is as follows:

(38) $$\gamma_{j_{i}}(r)=\Gamma (r,r-1)\gamma_{{\left(j-1\right)}_{i}}(r)+\lambda (r,r-1)w_{{\left(j-1\right)}_{i}}(r)-K_{j_{i}}(r){\tilde{Y}}_{j_{i}}(r|r-1)$$

Equation (38) simplifies to become the following:

(39) $$\gamma_{j_{i}}(r)=s_{j_{i-1}}(r)+q_{j_{i-1}}(r)-K_{j_{i}}(r){\tilde{Y}}_{j_{i}}(r|r-1)\;,$$

Take the probability density function and characteristic function on both sides of Equation (39), we can get the following:

(40) $$\sum_{L=1}^{m}(e^{jt\sigma_{L}}p_{e_{j_{i}}(r)})=\left[\sum_{L=1}^{m}\left(e^{jt\sigma_{L}}p_{s_{{}_{{\left(j-1\right)}_{i}}}(r)}\right)\right]\left[\sum_{L=1}^{m}\left(e^{jt\sigma_{L}}p_{q_{{\left(j-1\right)}_{i}}(r)}\right)\right]e^{-jtK(r){\tilde{Y}}_{j_{i}}(r|r-1)}$$

Combine the two lemmas given in Section 3 and Equation (39), and there is the following:

(41) $$\psi_{\gamma_{j_{i}}(r)}(t)=\psi_{s_{{\left(j-1\right)}_{i}}(r)}(t)\psi_{q_{{}_{{\left(j-1\right)}_{i}}}(r)}(t)e^{-jK(k+1){\tilde{Y}}_{j_{i}}(r|r-1)}$$

### 5.3. Establishment of Filter Performance Index 

Follow and repeat the process of Section 3.3 and Section 3.4, and the filter performance index can be described as follows:

(42) $$M_{j_{i}}(r)=M_{0}(r)+K_{j_{i}}^{T}(r)R_{j_{i}}(r)K_{j_{i}}(r)$$

Then $M_{j_{i}}$ can be rewritten as follows:

(43) $$M_{j_{i}}=\sigma_{j_{i}}\sigma_{j_{i}}^{T}+\sigma_{j_{i}}{\tilde{Y}}_{j_{i}}^{T}K_{j_{i}}^{T}c_{j_{i}}^{T}+\delta_{j_{i}}K_{j_{i}}{\tilde{Y}}_{j_{i}}\sigma_{j_{i}}^{T}+\delta_{j_{i}}K_{j_{i}}{\tilde{Y}}_{j_{i}}{\tilde{Y}}_{j_{i}}{}^{T}K_{j_{i}}{}^{T}\delta_{j_{i}}{}^{T}+K_{j_{i}}^{T}R_{j_{i}}K_{j_{i}}$$

Solve the first two-order partial derivative of Equation (43). The second-order partial derivative is greater than zero, and the filter gain matrix obtained by first-order partial is the optimal solution under the minimized performance index, $M_{j_{i}}$.

**Remark** **6.** *When the performance index has multiple poles, traditional solving methods may lead to local extremes and cause large errors, so we still introduce the fixed point equation as in Section 4.2.*

### 5.4. Establishment of Equation for Solving Gain Matrix $K_{j_{i}}(r)$

According to Section 4.2, construct a fixed point equation like $K_{j_{i}}(r)=d(K_{j_{i}}(r))$, to iteratively solve the filter gain matrix:

(44) $$K_{j_{i}t+1}=R_{j_{i}}^{-1}\delta_{j_{i}}^{T}\sigma_{j_{i}}{\tilde{Y}}_{j_{i}}^{T}+R_{j_{i}}^{-1}\delta_{j_{i}}\delta_{j_{i}}^{T}K_{j_{i}t}{\tilde{Y}}_{j_{i}}{\tilde{Y}}_{j_{i}}^{T}$$

where $t=0,1,2,\cdots ,T_{j_{i}}(r)$ represents the iteration steps of the fixed point method.

**Remark** **7.** *Initialize Equation (44) as $K_{j_{i}0}(r)=K_{{}_{{\left(j-1\right)}_{i}}}{}_{T_{j_{i}}(r)}^{}(r)$. In the fixed point equation, set a threshold β; if $K_{j_{i}t}^{}(r)$ satisfies Equation (45), the iterative process is ended.*

(45) $$\frac{{\left\|K_{j_{i}t}^{}(t)\right\|}_{2}-{\left\|K_{{\left(j-1\right)}_{i}}(t)\right\|}_{2}}{{\left\|K_{j_{i}t}^{}(t)\right\|}_{2}}\leq \beta$$

*Then Equation (35) can be rewritten as follows:*

(46) $${\hat{X}}_{j_{i}}(r|r)={\hat{X}}_{{\left(j-1\right)}_{i}}(r|r)+K_{j_{i}}{}_{T_{j_{i}}(r)}^{}(r){\tilde{y}}_{j_{i}}(r|r-1)$$

Until the L-th sensor transmits the information to the fusion center and completes filtering, the estimated value ${\hat{X}}_{L_{i}}(r|r)$ obtained at this time is the optimal estimated value of the sequential fusion method. 

## 6. Implementation Process of Sequential Characteristic Function Fusion Filtering Algorithm under Multi-Dimensional Observation

(1) Initialization:

(47) $${\hat{X}}_{0_{i}}(r|r)=\Gamma (r,r-1){\hat{X}}_{0_{i}}(r-1|r-1)$$

(2) Set arrival order:

At the r moment, assume that the measured value of the sensor transmitted to the fusion center via the wireless network is $Y_{1}(r),Y_{2}(r),\cdots ,Y_{i}(r),\cdots ,Y_{L}(r)$, and then we get the following:

(48) $${\hat{X}}_{{\left(j-1\right)}_{i}}(r|r)=E\{X_{{\left(j-1\right)}_{i}}(r)|X_{{\left(j-1\right)}_{i}}(0),Y_{{\left(j-1\right)}_{i}}(1),\cdots ,Y_{{\left(j-1\right)}_{i}}(r-1);Y_{1_{i}}(r),Y_{2_{i}}(r),\cdots ,Y_{{\left(j-1\right)}_{i}}(r)\}\;,$$

(3) Filter design:

Step 1: Design filters based on Equations (35) and (36).

(49) $${\hat{X}}_{j_{i}}(r|r)={\hat{X}}_{{\left(j-1\right)}_{i}}(r|r)+K_{{\left(j-1\right)}_{i}}(r){\tilde{Y}}_{{\left(j-1\right)}_{i}}(r|r-1)\;,$$

(50) $${\hat{Y}}_{j_{i}}(r|r-1)=h_{j_{i}}({\hat{X}}_{j_{i}}(r|r))$$

Step 2: Establish error recurrence equation according to Equation (39).

(51) $$\gamma_{j_{i}}(r)=s_{{\left(j-1\right)}_{i}}(r)+q_{{\left(j-1\right)}_{i}}(r)-K_{j_{i}}(r){\tilde{Y}}_{j_{i}}(r|r-1)$$

Step 3: Solve the error characteristic function propagation equation according to two lemmas.

(52) $$\psi_{\gamma_{j_{i}}(r)}(t)=e^{-jtK_{j_{i}}(r){\tilde{Y}}_{j_{i}}(r|r-1)}\psi_{s_{{}_{{\left(j-1\right)}_{i}}}(r)}(t)\psi_{q_{{\left(j-1\right)}_{i}}(r)}(t)$$

Step 4: Obtain the performance index function according to Equation (42).

(53) $$M_{j_{i}}(r)=M_{0}(r)+K_{j_{i}}^{T}(r)R_{j_{i}}(r)K_{j_{i}}(r).$$

Step 5: Establish the filter gain solving equation according to Equation (43).

(54) $$\frac{\partial M_{j_{i}}}{\partial K_{j_{i}}}=0,\frac{\partial^{2}M_{j_{i}}}{\partial K_{j_{i}}^{2}}>0$$

Step 6: Solve the filter gain matrix $K_{i_{t+1}}^{}(r)$ according to Equation (44).

(55) $$K_{j_{i}t+1}=R_{j_{i}}^{-1}\delta_{j_{i}}^{T}\sigma_{j_{i}}{\tilde{Y}}_{j}^{}{}_{i}+R_{j_{i}}^{-1}\delta_{j_{i}}^{T}\sigma_{j_{i}}K_{j_{i}t}{\tilde{Y}}_{j_{i}}{\tilde{Y}}_{j_{i}}^{T}$$

Step 7: Substitute the solved $K_{j_{i}}(r)$ into Equation (49).

(4) Repeat the above process.

Step 1: Obtain the state prediction value of the first sensor arriving at the fusion center

(56) $${\hat{X}}_{1_{i}}(r|r-1)=\Gamma (r,r-1){\hat{X}}_{1_{i}}(r-1|r-1)$$

Step 2: Solve the filter gain of the first sensor that reaches the fusion center $K_{1_{i}}(r){\tilde{Y}}_{1_{i}}(r|r-1)$.

Step 3: Calculate the state estimate value of the first sensor that reaches the fusion center.

(57) $${\hat{X}}_{1_{i}}(r|r)=\Gamma (r,r-1){\hat{X}}_{1_{i}}(r-1|r-1)+K_{1_{i}}(r){\tilde{Y}}_{1_{i}}(r|r-1)$$

Step 4: Take the first arrival state estimation value ${\hat{X}}_{1_{i}}(r|r)$ obtained in Step 3 as the second arrival state prediction value.

(58) $${\hat{X}}_{2_{i}}(r|r-1)={\hat{X}}_{1_{i}}(r|r)$$

After another round of cyclic Equations (56)–(58), the state estimation value of the second sensor that reaches the center can be obtained, denoted as ${\hat{X}}_{2_{i}}(r|r)$.

Step 5: Repeat the above steps, until all measurements in the $L\left(L\leq N\right)$ group are transmitted to the fusion center, the iteration ends. The corresponding estimated value is the optimal estimated value of the sequential characteristic function filtering. That is $\hat{X}(r|r)={\hat{X}}_{L_{i}}(r|r)$.

## 7. Simulation 

This paper uses three typical nonlinear systems to illustrate the effectiveness of the proposed three fusion methods. The first category is to imitate actual target tracking, the second category is from real industrial devices, and the third category is a general nonlinear model. Table 1 is used to present the application of three typical cases and the reasons why these cases were selected.

**Case 1:** Given a class of target tracking system, which is composed by

(59) $$\left[\begin{array}{c} X_{1}(r)\\ X_{2}(r)\\ X_{3}(r)\\ X_{4}(r)\\ X_{5}(r)\\ X_{6}(r) \end{array}\right]=\left[\begin{array}{cccccc} 1 & 0 & 0 & T & 0 & 0\\ 0 & 1 & 0 & 0 & T & 0\\ 0 & 0 & 1 & 0 & 0 & T\\ 0 & 0 & 0 & 1 & 0 & 0\\ 0 & 0 & 0 & 0 & 1 & 0\\ 0 & 0 & 0 & 0 & 0 & 1 \end{array}\right]\left[\begin{array}{c} X_{1}(r-1)\\ X_{2}(r-1)\\ X_{3}(r-1)\\ X_{4}(r-1)\\ X_{5}(r-1)\\ X_{6}(r-1) \end{array}\right]+\left[\begin{array}{c} w_{1}(r-1)\\ w_{2}(r-1)\\ w_{3}(r-1)\\ w_{4}(r-1)\\ w_{5}(r-1)\\ w_{6}(r-1) \end{array}\right]$$

(60) Y1(r)Y2(r)Y3(r)=X12(r)+X22(r)+X32(r)arcosX3(r)X12(r)+X22(r)+X32(r)arctanX2(r)X1(r)+v1(r)v2(r)v3(r)

where $X_{1},X_{2},X_{3},X_{4},X_{5},X_{6}$ represent the position and velocity on the x,y,z axes, and $Y_{1},Y_{2},Y_{3}$ respectively represent the radial distance between the target and sensors, and the two direction angles formed by the coordinate axis. 

In this case, three sensors are used to carry out the experiment. We simulate different measurement environments by setting different measurement noise covariance. The measurement noise variances of the three sensors are, respectively,$Q_{v1}\;=\;\mathrm{diag}([0.004,0.004,0.004])$, $Q_{v2}\;=\;\mathrm{diag}([0.003,0.003,0.004])$, $Q_{v3}\;=\;\mathrm{diag}([0.002,0.002,0.003])$. Set the process noise covariance as $Q_{w}\;=\;\mathrm{diag}([0.004,0.003,0.003,0.002,0.002,0.001])$ and initial conditions as $x(0)={\left[20,5,12,5,8,10\right]}^{T}$. Given objective characteristic function $\psi_{g}{(t)=e}^{-0.0005tIt^{T}}$ and filter weight function $R\left(k\right)=diag([5\times 10^{-5},4\times 10^{-5},3\times 10^{-5},3\times 10^{-5},2\times 10^{-5},2\times 10^{-5}])$ . Moreover, given a weight function $\Lambda (t)={\left[0.08e^{it\mu_{1}-tQ_{1}t^{T}},0.06e^{it\mu_{2}-tQ_{2}t^{T}},0.05e^{it\mu_{3}-tQ_{3}t^{T}}\right]}^{T}$ that can guarantee the filter performance parameters are bounded, where $\mu_{1}=0.0001,\mu_{2}=0.0001,\mu_{3}=0.00015$, $Q_{1}=0.0005I,Q_{2}=0.0004I,Q_{3}=0.0003I$, I is the unit matrix.

**Figure 5:**
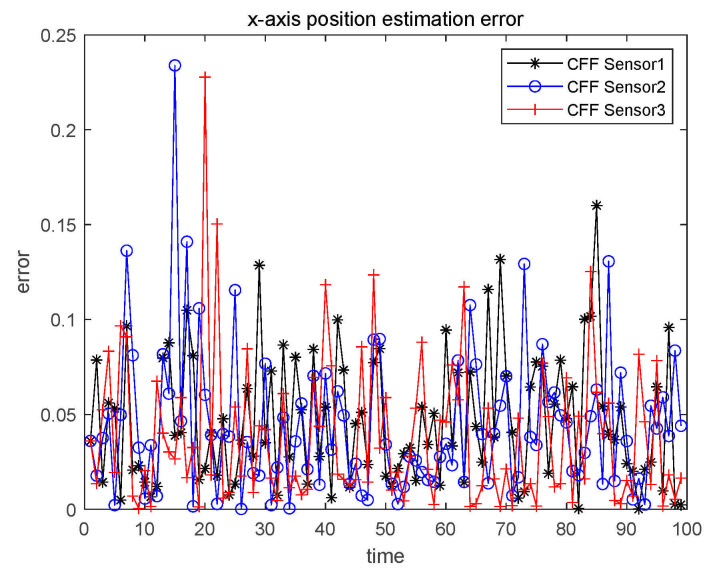
x1 single sensor estimation error.

We perform characteristic function filtering on the three sensors, respectively. In order to make the result analysis clearer, we only give the result graph of x1 in Case 1, and focus on it. The analysis of state x2–x6 is the same as x1, and the numerical results are all given in the table. The estimation error of x1 is shown in Figure 5.

Due to the random noise generated in the simulation experiment, we obtain the Monte Carlo average value of 100 times for the filtering result, and the mean square error is recorded in Table 2.

**Figure 6:**
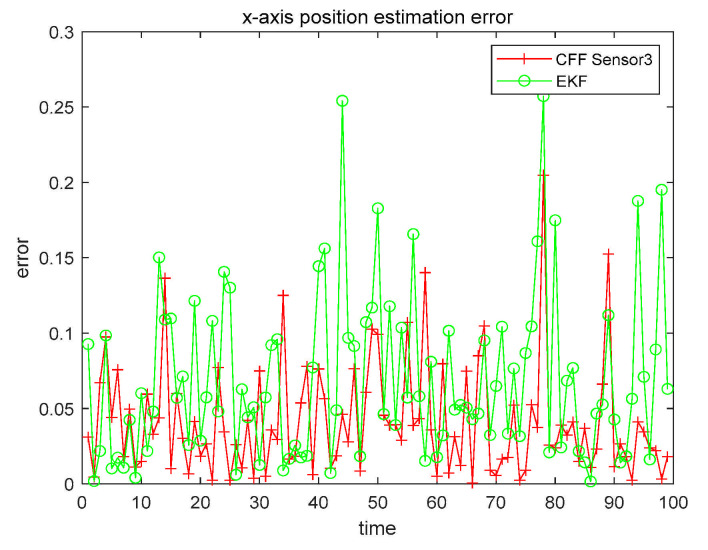
x1 CFF and Extended Kalman Filter (EKF) contrast error.

In Table 2, it can be seen that the accuracy of each sensor is different. Then select the highest precision sensor, and perform CFF and EKF, to further study the filtering effect of CFF in nonlinear systems, as shown in Figure 6.

From Figure 6, we can clearly see that the CFF filtering effect is better than EKF. To better analyze the results, we recorded estimation error in Table 3, and also recorded the accuracy improvement ratio of using the most accurate sensor for CFF, as compared to EKF. 

From the experimental results in Table 3, it can be analyzed that the effect of using CFF is better than that of EKF in nonlinear systems.

**Figure 7:**
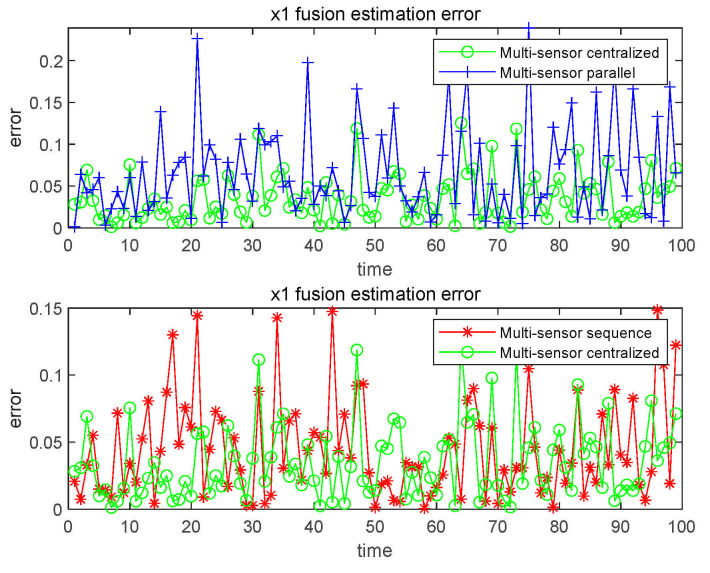
x1 fusion estimation error.

To further improve the estimation accuracy of CFF in nonlinear systems, we study the fusion method based on CFF. When using sequential fusion filtering method, the accuracy of the sensor is carried out, from low to high, to simulate the order in which sensors transmit information to the fusion center, as shown in Figure 7.

It can be directly observed from Figure 7 that the estimation accuracy of the centralized fusion method is significantly higher than that of the parallel fusion method, and higher than or even close to the sequential fusion method. To make the results more convincing, we also recorded the numerical results, as shown in Table 4.

From the data in Table 4, it can be concluded that the filtering effect of the three fusion methods is better than that of using only one sensor, and the centralized fusion method has the highest accuracy.

For space-moving targets, especially high-speed moving targets, when the target’s velocity increases, the target’s motion state will change, and the nonlinear characteristics of the system will also increase. In order to further explore the filtering effect of the fusion method in nonlinear system, we further study by changing the initial velocity and the given characteristic function, as shown in Table 5.

It can be analyzed from the data in Table 5 that, with the enhancement of system nonlinearity, the filtering effect of CFF is significantly better than that of EKF. At the same time, no matter which method of multiple sensor fusion, with the enhancement of nonlinearity, it is better than the filtering effect of using only a single sensor.

**Remark** **8.** *When conducting multiple sets of experiments, we only discussed the changes of the initial velocity and the target characteristic function in this case. This is because, when the velocity is very large, the measurement equation is almost a super-nonlinear equation, so the change of velocity can cause a very obvious change in the degree of system nonlinearity. We also tried to change μ and Q in the weight function Λ(t), but we found that the change has very little effect on the result. This is because the appearance of Λ(t) is to ensure that $M_{0}$ is bounded, so as long as $M_{0}$ is bounded, ans changes in μ and Q will not significantly affect the results.*

**Case 2:** Given a parameter identification system for a type of industrial device.

**Figure 8:**
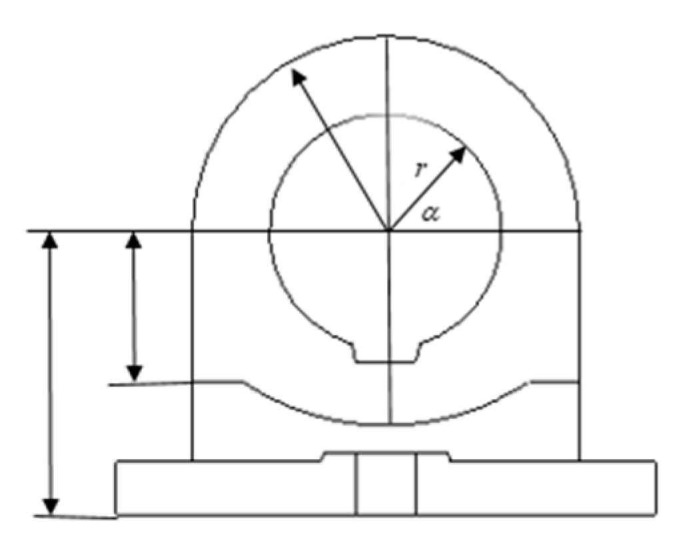
Industrial device model diagram.

In the actual measurement of industrial devices, as shown in Figure 8, the state equations are generally not complicated, but in order to obtain very accurate parts size and other parameters, the measurement equations are often very complicated and exhibit nonlinear characteristics. Therefore, multiple sensor fusion methods are usually considered to further improve the estimation accuracy of parameters, especially in nonlinear systems that require very high accuracy.

(61) $$\left[\begin{array}{l} X_{1}(r)\\ X_{2}(r) \end{array}\right]=\left[\begin{array}{cc} 1 & T\\ 0 & 1 \end{array}\right]\left[\begin{array}{l} X_{1}(r-1)\\ X_{2}(r-1) \end{array}\right]+\left[\begin{array}{l} w_{1}(r-1)\\ w_{2}(r-1) \end{array}\right]$$

(62) $$\left[\begin{array}{c} Y_{1}(r)\\ Y_{2}(r) \end{array}\right]=\left[\begin{array}{c} \sin(\alpha X_{1}(r))+\cos(\alpha X_{2}(r))\\ \sin(\beta X_{2}(r))+\cos(\beta X_{1}(r)) \end{array}\right]+\left[\begin{array}{c} v_{1}(r)\\ v_{2}(r) \end{array}\right]$$

where $X_{1},X_{2}$ are the parameters of industrial devices; $Y_{1},Y_{2}$ are the measurement parameters of industrial devices; and α and β are adjustable coefficients. Given that the measurement noise variances of the three sensors are respectively $Q_{v1}\;=\;\mathrm{diag}([0.004,0.004])$, $Q_{v2}\;=\;\mathrm{diag}([0.003,0.003])$, $Q_{v3}\;=\;\mathrm{diag}([0.002,0.003])$, set the process noise covariance as $Q_{w}\;=\;\mathrm{diag}([0.002,0.003])$ and initial conditions as $X(0)={\left[0.0002,0.0003\right]}^{T}$. The objective characteristic function is $\psi_{g}{(t)=e}^{-0.0005tIt^{T}}$, and the filter weight function $R\left(k\right)=diag([4\times 10^{-5},3\times 10^{-5}])$. Moreover, given a weight function $\Lambda (t)={\left[0.65e^{jt\mu_{1}-tQ_{1}t^{T}},0.05e^{jt\mu_{2}-tQ_{2}t^{T}}\right]}^{T}$ that can guarantee the filter performance parameters are bounded, where $\mu_{1}=0.0001,\mu_{2}=0.0001$, $Q_{1}=0.0005I,Q_{2}=0.0004I$, I is the unit matrix. 

**Figure 9:**
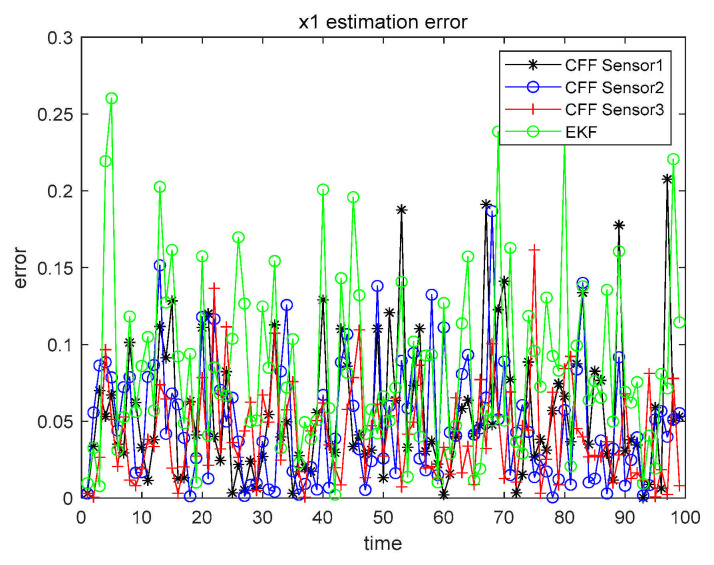
Case 2 x1 single sensor estimation error.

**Figure 10:**
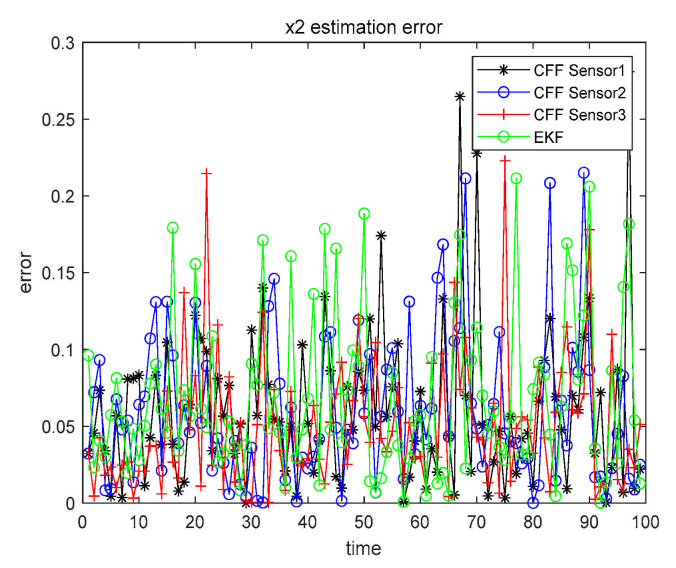
Case 2 x2 single sensor estimation error.

The detailed analysis steps are the same as in Case 1. Thus, in Cases 2 and 3, we did not give a very detailed description as in Case 1, but simplified the expression. When α=β=0.1, the three sensors perform CFF separately and, at the same time, perform EKF on the sensor with the highest accuracy. The results are shown in Figure 9 and Figure 10.

Similarly, in order to make the results more clearly presented, all numerical results are recorded in Table 6.

**Figure 11:**
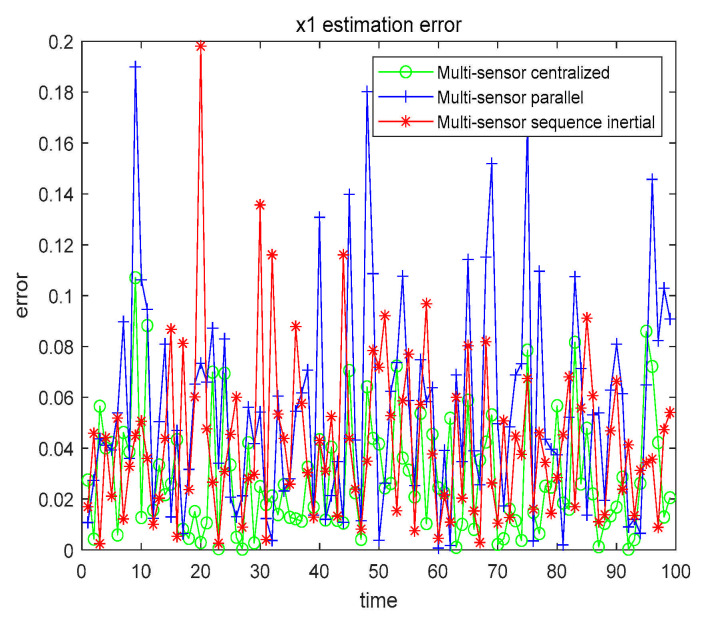
Case 2 x1 fusion estimation error.

**Figure 12:**
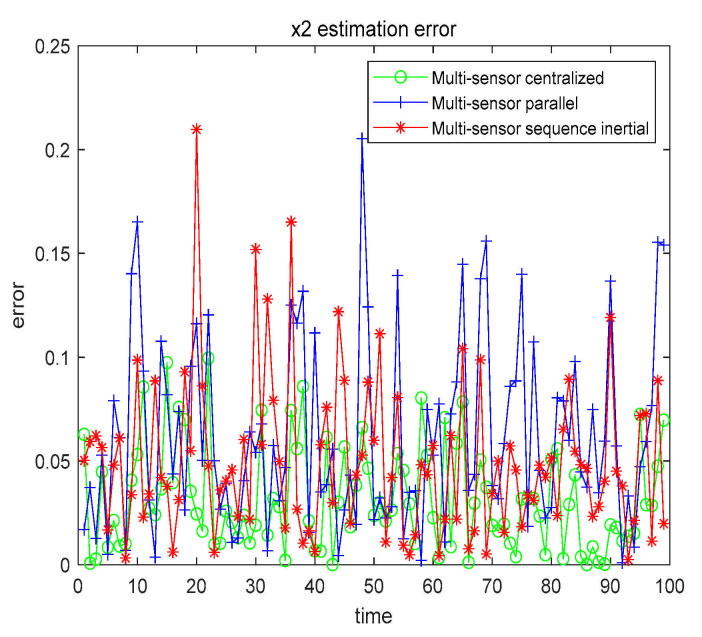
Case 2 x2 fusion estimation error.

From the data in Table 6, we can conclude that the filtering effect of CFF is significantly better than that of EKF. We also compared the three fusion methods based on CFF, as shown in Figure 11 and Figure 12. 

The numerical results are also compared with the estimated results of the single sensor with the highest accuracy. All numerical results are recorded in Table 7.

From Table 7, we can get that, no matter which fusion method, the filtering effect is better than that of using only a single sensor. In order to further explore the filtering effect of the fusion method in the nonlinear system, we further study by changing α, β, and the given characteristic function $\psi_{g}\left(t\right)$, as shown in Table 8.

From the data in the Table 8, it can be seen that, when the measurement model of the system is a more complex nonlinear model, the multi-sensor fusion algorithm based on CFF can obtain higher accuracy. At the same time, no matter which fusion method is used, the estimation accuracy is higher than that of only one sensor.

**Case 3:** Given a general nonlinear system

In case 3, the characteristic function fusion filtering algorithm is further extended to systems with weakly nonlinear state models. In order to better describe weak nonlinearity, we introduce trigonometric functions in the state equation. The general nonlinear model is shown as follows.

(63) $$\left[\begin{array}{c} X_{1}(r)\\ X_{2}(r) \end{array}\right]=\left[\begin{array}{c} 0.1X_{1}(r-1)+0.2X_{2}(r-1)\\ \sin(X_{1}(r-1))+\cos(X_{2}(r-1)) \end{array}\right]+\left[\begin{array}{c} w_{1}(r-1)\\ w_{2}(r-1) \end{array}\right]$$

(64) $$\left[\begin{array}{c} Y_{1}(r)\\ Y_{2}(r) \end{array}\right]=\left[\begin{array}{c} \sin(\alpha_{i}X_{1}(r))+\cos(\beta_{i}X_{2}(r))\\ X_{1}(r)+X_{2}(r) \end{array}\right]+\left[\begin{array}{c} v_{1}(r)\\ v_{2}(r) \end{array}\right]$$

where $X_{1},X_{2}$ are state vectors; $Y_{1},Y_{2}$ are measurement vectors; and $\alpha_{i}$ and $\beta_{i}$ are adjustable coefficients. Given that the measurement noise variances of the three sensors are respectively $Q_{v1}\;=\;\mathrm{diag}([0.001,0.001])$, $Q_{v2}\;=\;\mathrm{diag}([0.002,0.002])$, and $Q_{v3}\;=\;\mathrm{diag}([0.001,0.002])$, set process noise covariance as $Q_{w}\;=\;\mathrm{diag}([0.002,0.002])$ and initial conditions as $X(0)={\left[0.001,0.002\right]}^{T}$. The objective characteristic function is $\psi_{g}{(t)=e}^{-0.0005tIt^{T}}$, and filter weight function is $R\left(k\right)=diag\{3\times 10^{-5},3\times 10^{-5}\}$. Moreover, given a weight function $\Lambda (t)={\left[0.06e^{jt\mu_{1}-tQ_{1}t^{T}},0.03e^{jt\mu_{2}-tQ_{2}t^{T}}\right]}^{T}$ that can guarantee the filter performance parameters are bounded, where $\mu_{1}=0.002,\mu_{2}=0.003$, $Q_{1}=0.0005I,Q_{2}=0.0004I$, I is the unit matrix. 

**Figure 13:**
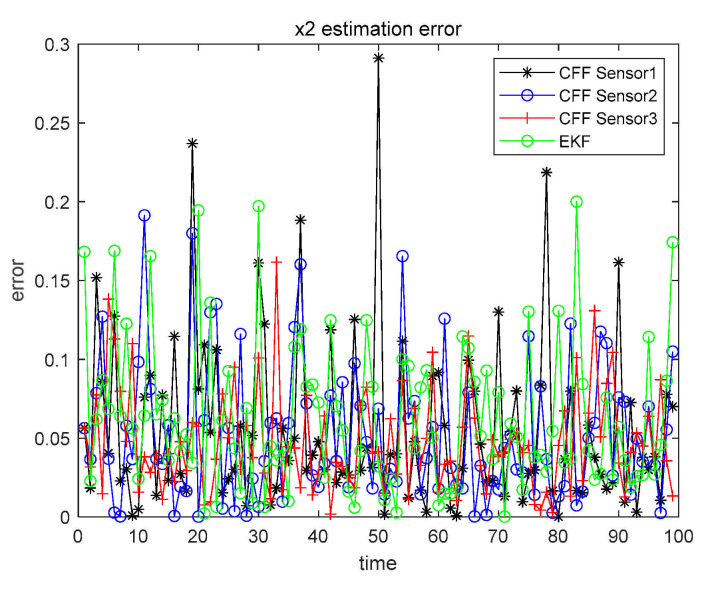
Case 3 x1 single sensor estimation error.

**Figure 14:**
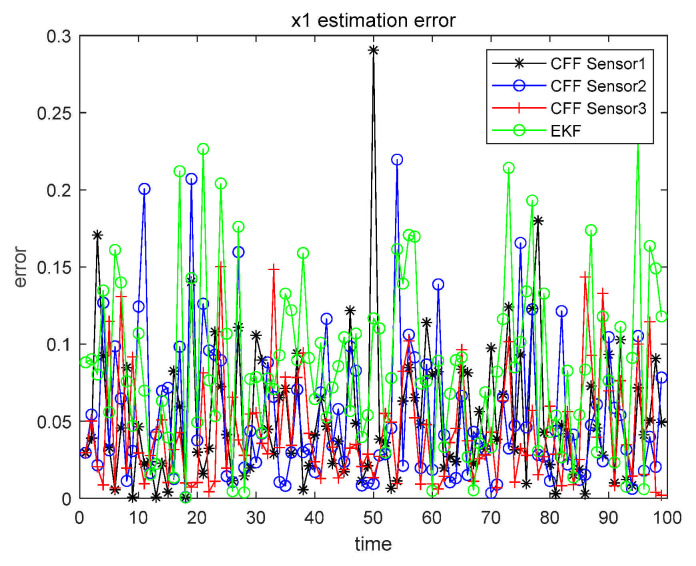
Case 3 x2 single sensor estimation error.

In Case 3, three sensors are also used for simulation, and the parameters of the three sensors are set as $\alpha_{1}=\beta_{1}=0.1$, $\alpha_{2}=\beta_{2}=0.2$, $\alpha_{3}=\beta_{3}=0.3$. The three sensors perform CFF separately and, at the same time, perform EKF on the sensor with the highest accuracy. The results are shown in Figure 13 and Figure 14. 

Similarly, all numerical results are recorded in Table 9.

**Figure 15:**
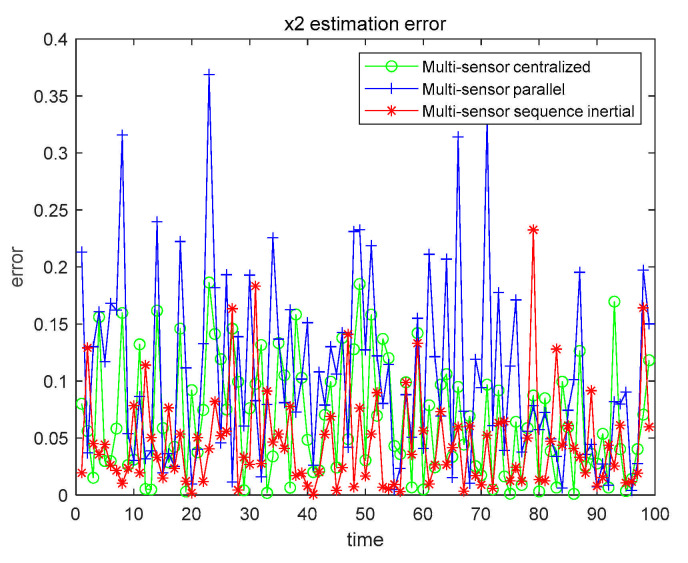
Case 3 x1 fusion estimation error.

**Figure 16:**
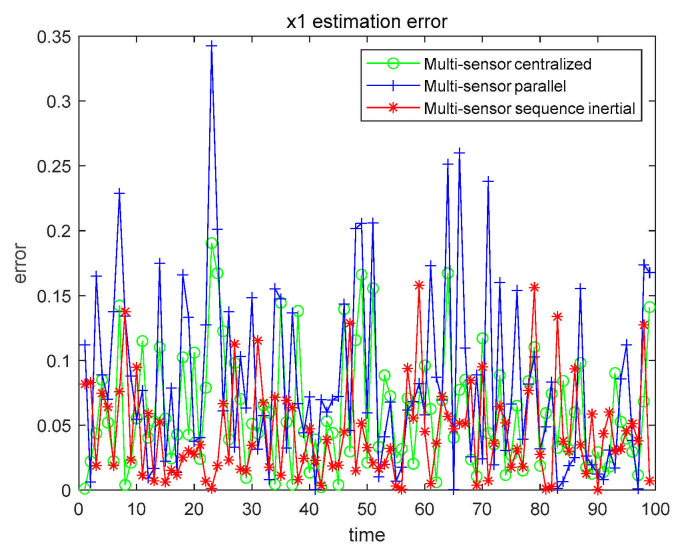
Case 3 x2 fusion estimation error.

It can be obtained from the data in Table 9 that, for general nonlinear equations, CFF can still obtain better filtering effects than EKF. Three fusion methods based on CFF are also compared, as shown in Figure 15 and Figure 16. Moreover, the numerical results are compared with the estimated results of the single sensor with the highest accuracy. All numerical results are recorded in Table 10.

From the data in the Table 10, it can be seen that, for the general nonlinear measurement equation, the multi-sensor fusion algorithm based on CFF can obtain higher accuracy. It also shows that three fusion methods based on CFF not only achieve good filtering effects in systems where the state is linear and measured as nonlinear, but also in systems where the state model is weakly nonlinear.

Generally speaking, in the actual system, according to different requirements, such as high precision, being easy to implement, or as close to the actual situation as possible, we can choose different fusion methods for state estimation.

## 8. Conclusions

In this study, three fusion filters were designed for a class of strong nonlinear measurement systems based on CFF, namely as centralized, parallel, and sequential. They were designed to meet the different needs of the systems, such as accuracy, being easy to implement, or matching with the actual environment. The performances of the three fusion filters are illustrated by three typical cases. Since EKF is the most popular method in the nonlinear system, we compared CFF with EKF. The results show that, under the same conditions, all filters show good performance, but the performance of the three fusion filters we designed is better than the most popular EKF performance, respectively. The fundamental reason is that the performance of CFF is better than that of EKF: (1) On the basic of introducing new performance indicators, the proposed CFF avoids the large truncation error caused by Taylor expansion like EKF. (2) CFF relaxes the requirements for the statistical characteristics of the error, and EKF’s requirement for the ideal white noise of the error is replaced by the characteristic function of the error in the CFF.

Motivated by the results of this paper, we need to further think about the following issues: (1) These results were all established under the condition that the characteristic function of the target was given. In complex situations, how to make an accurate characteristic function or whether the characteristic function of the actual system can be obtained through a certain solution method still needs further study. (2) We imitated the EKF to solve the weakly nonlinear state, but in the face of a large number of strongly nonlinear state models, how to design a CFF model suitable for strong nonlinearity is still worth studying. (3) The fusion methods in this paper were all established under the condition that the error characteristic functions were independent. How to design the corresponding CFF when the error characteristic functions are related is worthy of further study.

## Figures and Tables

**Table 1:** Typical cases.

Case	Typical Case Application	Reason for Selecting This Case
Case 1	Space moving target tracking system	Generally, the measurement of space targets is carried out in the polar coordinate system, and it is usually necessary to perform a unified transformation in the rectangular coordinate system before further data processing. The method based on characteristic function in this paper, avoids the error caused by conversion and also avoids the rounding error caused by the linearization process.
Case 2	Industrial device measurement system	For high-precision industrial devices, the measurement equations must be complicated, and may be super-nonlinear equations. In order to obtain very accurate parameter results, the coordinated measurement of multiple sensors can be used.
Case 3	General nonlinear system	In order to make the fusion method universal, a general case is used to demonstrate the effectiveness of the proposed methods.

**Table 2:** Mean square error.

Sensors	CFF Sensor1	CFF Sensor2	CFF Sensor3
Mean square error	0.07864	0.07953	0.06221

CFF, characteristic function filter.

**Table 3:** Case 1 CFF and EKF error comparison.

State Value	CFF Sensor1	CFF Sensor2	CFF Sensor3 (best)	EKF	CFF Accuracy Increase Ratio
x1	0.04892	0.04762	0.04692	0.04954	5.5839%
x2	0.05691	0.05572	0.05120	0.06452	26.0156%
x3	0.06618	0.06650	0.05993	0.06732	12.3311%
x4	0.07436	0.07894	0.07642	0.07991	4.8687%
x5	0.09174	0.09501	0.09402	0.09853	4.7969%
x6	0.10972	0.10150	0.10618	0.11985	12.8744%

**Table 4:** Case 1 fusion method error comparison.

State Value	CFF Sensor3	Centralized	Accuracy Increase Ratio	Parallel	Accuracy Increase Ratio	Sequence	Accuracy Increase Ratio
x1	0.04692	0.04017	16.8034%	0.04313	8.7874%	0.04271	9.8572%
x2	0.05120	0.04268	19.9625%	0.04835	5.8945%	0.04352	17.6471%
x3	0.05993	0.04605	30.1412%	0.05471	9.5412%	0.04782	25.3241%
x4	0.07642	0.06484	17.8593%	0.06915	10.5134%	0.06509	17.4067%
x5	0.09402	0.07812	20.3533%	0.09025	4.1773%	0.08504	10.5597%
x6	0.10618	0.08470	25.3601%	0.09724	9.1937%	0.08612	23.2931%

**Table 5:** Case 1 variable parameter error comparison.

x4(0)	x5(0)	x6(0)	$\psi_{g}\left(t\right)$	CFF Mean Square Error	EKF Mean Square Error	CFF Accuracy Increase Ratio	Centralized Mean Square Error	Centralized Accuracy Increase Ratio
5	8	10	$e^{-0.0002tIt^{T}}$	0.0621	0.0663	6.7633%	0.0637	2.5765%
5	8	10	$e^{-0.0010tIt^{T}}$	0.0823	0.0899	9.2345%	0.0864	4.9818%
10	15	20	$e^{-0.0015tIt^{T}}$	0.0876	0.1004	14.6119%	0.0995	13.5845%
15	20	25	$e^{-0.0020tIt^{T}}$	0.1027	0.1273	27.9533%	0.1192	16.0662%

**Table 6:** Case 2 CFF and EKF error comparison.

State Value	CFF Sensor1	CFF Sensor2	CFF Sensor3	EKF	CFF Accuracy Increase Ratio
x1	0.0678	0.0647	0.0702	0.0815	25.9660%
x2	0.0739	0.0698	0.0721	0.0846	21.2034%

**Table 7:** Case 2 fusion method error comparison.

State Value	CFF Sensor (best)	Centralized	Accuracy Increase Ratio	Parallel	Accuracy Increase Ratio	Sequence	Accuracy Increase Ratio
x1	0.0647	0.0619	4.5234%	0.0632	2.3734%	0.0621	4.1868%
x2	0.0698	0.0674	3.5608%	0.0686	1.7492%	0.0679	2.7982%

**Table 8:** Case 2 variable parameter error comparison.

α	β	$\psi_{g}\left(t\right)$	CFF Mean Square Error	EKF Mean Square Error	CFF Accuracy Increase Ratio	Centralized Mean Square Error	Centralized Accuracy Increase Ratio
0.05	0.05	$e^{-0.0001tIt^{T}}$	0.0583	0.0641	9.9485%	0.0557	4.6678%
0.5	0.5	$e^{-0.0005tIt^{T}}$	0.0624	0.0739	18.4295%	0.0571	9.2819%
1	5	$e^{-0.0020tIt^{T}}$	0.1062	0.1294	21.8456%	0.0913	16.3198%
5	10	$e^{-0.0050tIt^{T}}$	0.1375	0.1782	29.6000%	0.1149	19.6693%

**Table 9:** Case 3 CFF and EKF error comparison.

State Value	CFF Sensor 1	CFF Sensor 2 (best)	CFF Sensor 3	EKF	CFF Accuracy Increase Ratio
x1	0.0963	0.0887	0.0923	0.1032	16.3472%
x2	0.0805	0.0761	0.0892	0.0995	30.7490%

**Table 10:** Case 3 fusion method error comparison.

State Value	CFF Sensor (best)	Centralized	Accuracy Increase Ratio	Parallel	Accuracy Increase Ratio	Sequence	Accuracy Increase Ratio
x1	0.0887	0.0721	23.0236%	0.0867	2.3068%	0.0757	17.1731%
x2	0.0761	0.0718	5.9889%	0.0741	2.6990%	0.0724	5.1105%
